# Potential of Aligned Electrospun PLGA/SIS Blended Nanofibrous Membrane for Tendon Tissue Engineering

**DOI:** 10.3390/polym15102313

**Published:** 2023-05-15

**Authors:** Kihoon Kim, Hyosung Kim, Sunhee Do, Hwiyool Kim

**Affiliations:** 1 Department of Surgery, College of Veterinary Medicine, Konkuk University, Seoul 05029, Republic of Korea; chesvil@naver.com; 2 Department of Clinical Pathology, College of Veterinary Medicine, Konkuk University, Seoul 05029, Republic of Korea; w6373515@naver.com (H.K.); shdo@konkuk.ac.kr (S.D.)

**Keywords:** electrospinning, NIH-3T3, poly-d,l-lactide-co-glycolide, scaffold, small intestinal submucosa, tendon

## Abstract

Tendons are responsible for transmitting mechanical forces from muscles to bones for body locomotion and joint stability. However, tendons are frequently damaged with high mechanical forces. Various methods have been utilized for repairing damaged tendons, including sutures, soft tissue anchors, and biological grafts. However, tendons experience a higher rate of retear post-surgery due to their low cellularity and vascularity. Surgically sutured tendons are vulnerable to reinjury due to their inferior functionality when compared with native tendons. Surgical treatment using biological grafts also has complications such as joint stiffness, re-rupture, and donor-site morbidity. Therefore, current research is focused on developing novel materials that can facilitate the regeneration of tendons with histological and mechanical characteristics similar to those of intact tendons. With respect to the complications in association with the surgical treatment of tendon injuries, electrospinning may be an alternative for tendon tissue engineering. Electrospinning is an effective method for fabrication of polymeric fibers with diameters ranging from nanometers to micrometers. Thus, this method produces nanofibrous membranes with an extremely high surface area-to-volume ratio, which is similar to the extracellular matrix structure, making them suitable candidates for application in tissue engineering. Moreover, it is possible to fabricate nanofibers with specific orientations that are similar to those of the native tendon tissue using an adequate collector. To increase the hydrophilicity of the electrospun nanofibers, natural polymers in addition to synthetic polymers are used concurrently. Therefore, in this study, aligned nanofibers composed of poly-d,l-lactide-co-glycolide (PLGA) and small intestine submucosa (SIS) were fabricated using electrospinning with rotating mandrel. The diameter of aligned PLGA/SIS nanofibers was 568.44 ± 135.594 nm, which closely resembles that of native collagen fibrils. Compared to the results of the control group, the mechanical strength exhibited by the aligned nanofibers was anisotropic in terms of break strain, ultimate tensile strength, and elastic modulus. Elongated cellular behavior was observed in the aligned PLGA/SIS nanofibers using confocal laser scanning microscopy, indicating that the aligned nanofibers were highly effective with regard to tendon tissue engineering. In conclusion, considering its mechanical properties and cellular behavior, aligned PLGA/SIS is a promising candidate for tendon tissue engineering.

## 1. Introduction

A tendon is a densely arranged connective tissue capable of transmitting tensile forces from the muscle to the bone and is aligned parallel to its long axis [1]. Tendons are frequently injured when subjected to high mechanical loads [2]. Because the intrinsic regenerative capacity of tendons is limited due to their low cellularity and vascularity [3,4], damage to the tendon may result in severe pain and an inability to bear weight if not properly managed [5]. 

Various surgical management techniques involving sutures and soft tissue anchors have been used to treat tendon injuries [6]. However, surgically recovered tendons are vulnerable to reinjury because of their inferior functionality when compared with native tendons [4,6]. Surgical treatment with biological grafts is also often performed; however, such surgical options have complications such as joint stiffness, re-rupture, and donor-site morbidity [2,6]. Thus, current research in the field of tendon tissue engineering is focused on developing novel materials to facilitate the regeneration of tendons with histological and mechanical characteristics similar to those of uninjured tendons [7,8].

With respect to the aforementioned limitations associated with the surgical management of tendon injuries, electrospinning may be a promising alternative for tendon tissue engineering [9]. Electrospinning is an effective method that can be used to fabricate polymeric fibers with diameters ranging from nanometers to micrometers [10]. Thus, this method generates nanofibrous membranes (NFM) with an extremely high surface area-to-volume ratio, which is similar to the extracellular matrix (ECM) structure, making them suitable candidates for application in tissue engineering [11]. Moreover, it is possible to fabricate nanofibers with specific orientations that are similar to those of the native tendon tissue using an adequate collector during the electrospinning process [4,12]. Attempts have been made to obtain aligned nanofibers for various purposes. Aviss et al. produced aligned electrospun poly-d,l-lactide-co-glycolide (PLGA) nanofibers for skeletal muscle regeneration [13], while Subramanian et al. fabricated aligned electrospun PLGA- polycaprolactone (PCL) nanofibers for neural regeneration [14]. However, the hydrophobic features of synthetic polymers are often unsuitable for initial cell attachment [15]. In contrast to synthetic polymers, the hydrophilic properties of natural polymers, such as collagen, chitosan, and alginate, are more favorable for cell adhesion [15,16,17]. Over the past few decades, numerous studies have focused on the fabrication of hybrid scaffolds using synthetic and naturally derived polymers for both mechanical stability and biocompatibility for tissue engineering [16,18,19,20]. 

The small intestine submucosa (SIS), which originates from the submucosa of the porcine intestine, is one of the most widely used collagenous ECMs. It is primarily composed of type 1 and 2 collagen fibers (more than 90% of the entire collagen content). It also contains various cytokines, including glycosaminoglycans, heparins, transforming growth factor-β, insulin-like growth factor-1, epidermal growth factor, and vascular endothelial growth factor as well as chondroitin sulfates, fibronectins, and hyaluronic acids [21,22]. These cytokines are known to improve cellular attachment, migration, proliferation, and differentiation [23]. Several studies have used SIS as a blending material for hybridization with PCL [24,25]. In our previous study, we developed a novel scaffold of electrospun PLGA and SIS blended nanofibrous membranes and identified their potential for wound healing in an in vitro study [26]. However, these novel scaffolds were in non-woven web forms, and the mechanical strength of random nanofibers was generally poor [27]. Moreover, considering that most of the native ECMs, including tendons, have a defined or regularly oriented architecture, which is important for tissue function, random nanofibers are unsuitable for tendon tissue engineering [27,28]. 

The primary objective of this study was to investigate the potential use of aligned electrospun PLGA/SIS nanofibers in tendon tissue engineering. The aligned electrospun PLGA/SIS blended nanofibers were characterized using scanning electron microscopy (SEM), water contact angle (WCA), mechanical strength, cellular morphology, attachment, and proliferation. 

## 2. Materials and Methods

### 2.1. Materials

PLGA (50:50; ester terminated; mol. wt, 24,000–38,000 Da), Dulbecco’s modified Eagle’s medium (DMEM), fetal bovine serum (FBS), antibiotics (penicillin–streptomycin), and trypsin-ethylenediaminetetraacetic acid (EDTA) were supplied by Sigma (St. Louis, MO, USA). Phosphate-buffered saline (PBS) was purchased from Gibco (Carlsbad, CA, USA). 1,1,1,3,3,3-Hexafluoro-2-propanol (HFIP) was provided by Oakwood, Inc. (Estill, SC, USA). The phalloidin-iFluor 594 reagents (ab176757) used for F-actin staining were purchased from Abcam (Cambridge, MA, USA). 

### 2.2. Preparation of SIS Powder

SIS powder was prepared using a commercial pig, as previously described [29]. Briefly, SIS was obtained after the mechanical removal of the tunica mucosa, tunica muscularis externa, and tunica serosa layers using a scalpel blade. After washing with saline, the SIS was placed in 0.1% peracetic acid for 2 h for decellularization. The SIS was then washed several times with PBS and distilled water (DW). The SIS was lyophilized at −55 °C with a freeze-dryer and subsequently cryomilled using a freezer mill (6860; SPEX Inc., Metuchen, NJ, USA). SIS powder was not soluble in either water or HFIP. Therefore, it (1 wt% concentration) was digested with 0.1% pepsin, neutralized with NaOH, and lyophilized before electrospinning.

### 2.3. Fabrication of Random and Aligned Nanofibrous Mats (NFMs)

Random electrospun NFMs were obtained from aluminum foil fixed on a flat metal collector [26]. A rotating metal collector was used to fabricate uniaxially aligned nanofibers. The metal collector was rotated at a rate of 1000 rpm to improve the alignment of the nanofibers while keeping the other operating parameters constant. To fabricate the electrospun scaffolds, PLGA was dissolved at 25 wt% in HFIP, while a blended solution of PLGA/SIS was prepared by mixing 25 wt% PLGA and 1 wt% SIS in HFIP. Random and aligned NFMs were fabricated using each solution. All electrospinning experiments were performed at room temperature. The randomly oriented and aligned NFMs were stored in a vacuum desiccator until further use. 

### 2.4. Scanning Electron Microscopy

The morphology of the randomly oriented and aligned NFMs was identified using a scanning electron microscope (SEM; S-4800, Hitachi, Chiyoda, Japan) operating at an accelerating voltage of 5 kV. Five distinct random regions within the scaffold were selected for sample preparation. Platinum was sputtered onto the surface of each dried sample for 100 s at a current of 10 mA and continuously imaged. The diameter and orientation of the nanofibers were measured using ImageJ software (version 1.54d, NIH, Bethesda, MD, USA). 

### 2.5. Water Contact Angle

The water contact angles (WCA) of both the random and aligned NFMs were measured using a WCA analyzer (DSA 100, Krüss GmbH, Hamburg, Germany). Briefly, 2 µL deionized water was placed in the middle of each sample, and the water contact angle was measured using the sessile drop method. Each sample was measured at more than three different points, and the average was determined from at least five separate runs. 

### 2.6. Mechanical Properties

To investigate the effect of nanofiber alignment on the mechanical properties, the tensile strength of the NFMs was measured using a universal testing device with a 10,000 N load cell at a loading rate of 15 mm/min (AGS-X, Shimadzu, Kyoto, Japan). The mechanical properties of the PLGA and PLGA/SIS NFMs were identified in wet state. Each sample was cut into a 0.5 mm × 30 mm × 0.2 mm sheet of scaffold material for the measurement of the mechanical properties, including elastic modulus, ultimate tensile strength, and break strain. The average value of each parameter was measured for six specimens of each scaffold.

### 2.7. Cell Culture of NIH-3T3 Cells

NIH-3T3 cells were provided by S. Do (Veterinary Clinical Pathology, Konkuk University, Seoul, Korea). The NIH-3T3 cells were subcultured in DMEM containing 10% FBS and penicillin/streptomycin (100 μg/mL). The medium was changed every three days, and the cultures were placed in a cell culture incubator at 37 °C with 5% CO_2_. Once the cells reached 80–90% confluence, they were trypsinized, removed from the culture flask, and passaged at a 1:3 ratio. Passages 4–5 were used in this study.

### 2.8. Cell Attachment and Proliferation 

The effects of the hydrophilicity and alignment of the nanofibers on cell attachment and proliferation were evaluated by seeding NIH-3T3 cells onto random or aligned electrospun PLGA and SIS NFMs. Empty wells of the 96-well plate were used as background control. Cells were seeded in each well at a concentration of 5 × 10^3^ cells and placed in a humidified incubator for 7 days. During the culture period, the WST assay was performed on days 1, 3, and 7. After the removal of the medium from each well, 100 µL of the medium was added to each well along with 10 µL of WST-1 reagent. The plates were kept at 37 °C for 1 h to allow the WST reaction to occur. The plates were then placed into a microplate reader (Sunrise™, TECAN, Lyon, France) to read the absorbance at 450 nm to determine the amount of Formazan dye, which directly reflects the number of metabolically active viable cells.

### 2.9. Immunofluorescence Staining 

To evaluate the effect of scaffold architecture on cellular conformation, the cellular behavior on the aligned PLGA/SIS NFM was compared to that on the random PLGA NFM by labeling actin microfilaments with Fluorescein Isothiocyanate (FITC)-phalloidin. Immunofluorescence staining was performed to investigate the morphology and alignment of the NIH-3T3 cells. After aspirating the medium with special care to avoid dislodging the cells, the cells were rinsed once with PBS. The cells were then fixed in formalin (4% formaldehyde in PBS) at room temperature for 30 min. After removing the fixation solution, the cells were rinsed three times in PBS, permeabilized with 0.1% Triton X-100 for 5 min, and washed with PBS again. F-actin filaments were labeled with phalloidin-iFluor 594 (Abcam, Boston, MA, USA). Finally, the samples were counterstained with 4′,6-diamidino-2-phenylindole (DAPI) (Vectashield Mounting Medium; Vector Laboratories, Burlingame, CA, USA) to label the cell nuclei. 

### 2.10. Confocal Laser Scanning Microscope (CLSM)

To investigate the topography-induced behavior of NIH-3T3 cells, their morphology on both random and aligned scaffolds was evaluated using a confocal laser scanning microscope (LSM-800, Carl-Zeiss, Oberkochen, Germany). The gain/offset settings for the detectors were optimized to minimize the background noise. The contrast and brightness of digital images were optimized using Adobe Photoshop (Adobe Systems, San Jose, CA, USA). The scaffolds were fixed in 2.5% paraformaldehyde for 30 min at different time points (1st, 3rd, and 7th day). 

### 2.11. Statistical Analysis

The data were summarized and analyzed using statistical software (SPSS, version 23.0). Multi-way analysis of variance tests were conducted, and *p* values less than 0.05 were considered significant. All data were represented in the form of mean ± standard deviations (SD).

## 3. Results

### 3.1. Morphology of Aligned Nanofibrous Membrane

**Figure 1:**
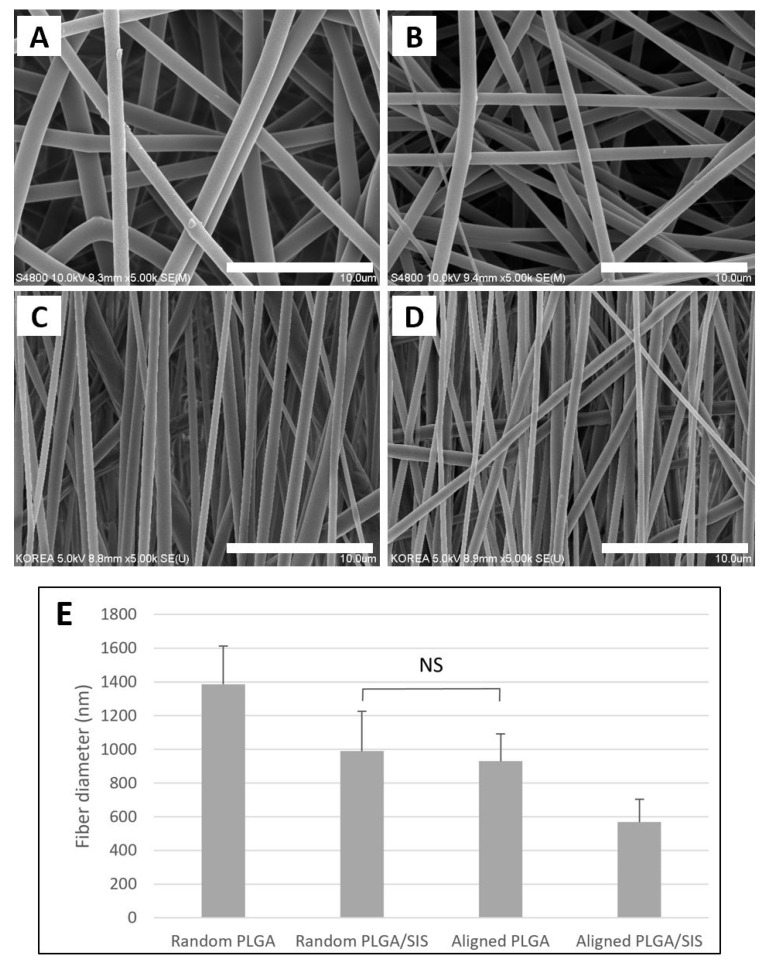
Scanning electron microscope (SEM) images of the nanofibrous scaffolds consisting of (**A**) random poly-d,l-lactide-co-glycolide (PLGA), (**B**) aligned PLGA, (**C**) random PLGA/small intestine submucosa (SIS), and (**D**) aligned PLGA/SIS and (**E**) the mean fiber diameter of different nanofibrous scaffolds (Scale bar = 10 μm). The mean fiber diameters of all the nanofibrous scaffolds were significantly different from each other, except between random PLGA/SIS and aligned PLGA (*p* < 0.05), NS: no significant difference.

Morphological differences between the random or aligned electrospun PLGA and PLGA/SIS NFMs were observed using SEM. As shown in Figure 1, the fibers were randomly arranged on the flat collector (Figure 1A,B). In comparison, although the alignment of every fiber was not perfectly oriented in the same direction, the fibers obtained on the rotating disc collector displayed a distinctly aligned longitudinal morphology (Figure 1C,D). The diameters (nm) of random and aligned PLGA NFMs were 1387.45 ± 225.88 nm and 929.34 ± 236.943 nm, respectively, while the diameters of the random and aligned PLGA/SIS NFMs were 989.34 ± 162.994 nm and 568.44 ± 135.594 nm, respectively. When the orientation of the nanofibers was the same, the addition of SIS significantly decreased the diameters (*p* < 0.05). This is assumed to result from the increased electrical conductivity due to the incorporation of SIS into PLGA [25,26]. However, under the same NFM composition, the diameter of the aligned nanofibers decreased significantly compared to that of the random nanofibers (*p* < 0.05). This was because the rotating mandrel spun faster than the speed at which nanofibers were produced; thus, physically stretching the nanofibers in the rotating direction of the mandrel [30]. However, there was no significant difference between the diameter (mn) of the aligned PLGA nanofibers and that of the random PLGA/SIS nanofibers. 

### 3.2. Water Contact Angle (WCA)

**Figure 2:**
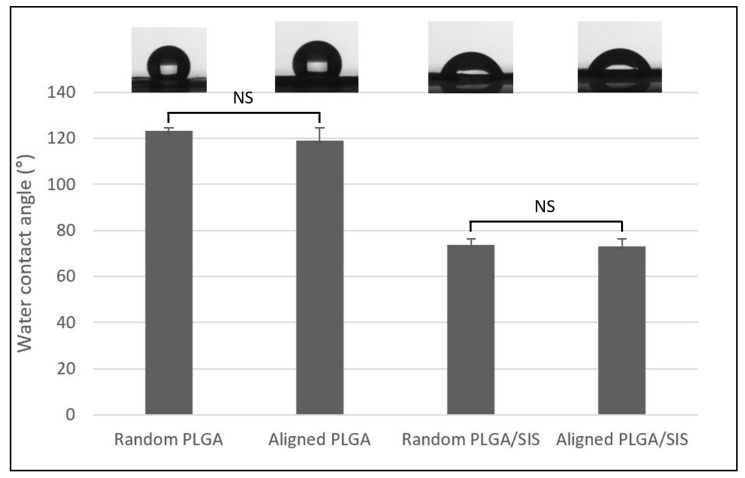
Variations in the water contact angle of the random and aligned PLGA and SIS nanofibrous membranes (NFMs). NS: no significant difference.

Because cells adhere and migrate more efficiently on the surface under appropriate hydrophilic conditions, we evaluated the static contact angle of deionized water on the random and aligned NFMs (Figure 2). As a result, the WCA of random PLGA, aligned PLGA, random PLGA/SIS, and aligned PLGA/SIS NFMs were 122.56 ± 3.56°, 119.52 ± 6.52°, 74.12 ± 4.61°, and 73.87 ± 5.2°, respectively. As expected, the surface of the random and aligned PLGA/SIS NFMs was more hydrophilic than that of the random and aligned PLGA NFMs, due to the addition of SIS to PLGA [26]. According to a previous study, a WCA of aligned nanofibers is lower than that of random nanofibers due to the smaller pore size of aligned scaffolds [14]. However, a significant difference in the WCA of the aligned NFMs was not identified when compared to the random NFMs. We speculate the reason for this result is that the alignment of the nanofibers was not sufficient to cause a difference in the WCA. 

### 3.3. Mechanical Properties

**Figure 3:**
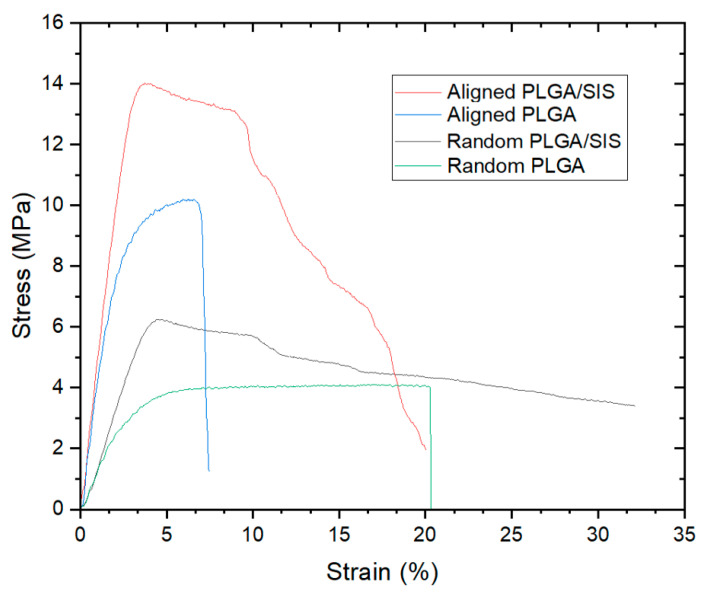
Representative curve of the random and aligned NFMs.

To clarify the effects of the incorporation of SIS into the blended nanofibers and the alignment of the nanofibers on the mechanical properties of the electrospun nanofibers, a uniaxial tensile strength test was performed. Consequently, the different orientations of the nanofibers resulted in anisotropic mechanical properties. In the current study, the break strain, tensile strength, and elastic modulus of aligned PLGA/SIS NFMs were higher than those of the other NFMs (*p* < 0.05). We found that the incorporation of SIS into the blended nanofibers altered the mechanical behavior from brittle to ductile (Figure 3). Considering that weak mechanical strength exhibits brittle mechanical behavior, the incorporation of SIS into pure PLGA strengthened the scaffolds, which is consistent with the results of a previous study [26]. However, the ultimate tensile strength of the aligned nanofibers was improved compared to that of the random nanofibers, regardless of the incorporation of SIS (*p* < 0.05). It is believed that the direction of the nanofibers enhanced mechanical properties, resulting in superior mechanical performance of the aligned nanofibers than that of random nanofibers. This was outlined in two previous studies that described the mechanical properties in association with the angle and of the stretching direction and the fiber orientation [31,32].

The tensile strengths of the human patella and rotator tendons are 5–65 and 14–45 MPa, respectively. Therefore, ultimate tensile strength of the aligned PLGA/SIS nanofibers observed in this study was higher than the lower value of the tensile strength of each of the aforementioned actual anisotropic tissues. Moreover, the maximum strength and tensile modulus of Artelon^®^ (SportMesh^TM^ Artimplant AB, Frölunda, Sweden), a commercial patch used for soft tissue reinforcement, including tendon, are 11.86 MPa and 14.25 MPa, respectively [33], which are lower than the maximum strength and tensile modulus of the aligned PLGA/SIS nanofibers used in this study. The elastic modulus, ultimate tensile strength, and break strain values are listed in Table 1.

### 3.4. Comparison of the Morphology of NIH-3T3 Cells on Random and Aligned NFMs

**Figure 4:**
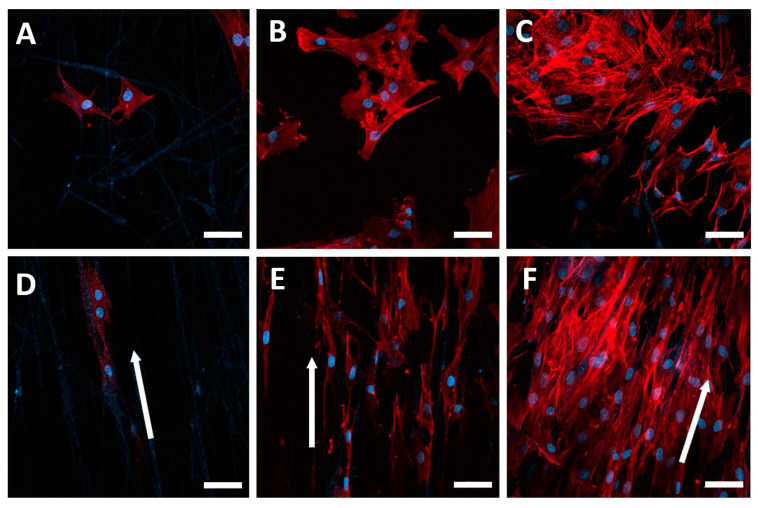
Representative images of the cellular behavior of random PLGA and aligned PLGA/SIS NFMs after 1, 3, and 7 days of culture. (**A**–**C**) Randomly oriented cell growth on random PLGA NFMs after 1, 3, and 7 day of culture. (**D**–**F**) Regular cell growth on aligned PLGA/SIS NFMs after 1, 3, and 7 day of culture (white arrow: direction of cell growth). F-actin was labelled with phalloidin-iFluor 594 (red); cell nuclei were counterstained with DAPI (blue). The scale bar for confocal fluorescence micrographs is 50 µm.

The alignment of nanofibers is crucial in tissue engineering. Cells in natural tissues are often regularly oriented [25]. The morphology of the NIH-3T3 cells on both random PLGA and aligned PLGA/SIS scaffolds was identified using CLSM on days 1, 3, and 7 after seeding. We found that cells on both random and aligned NFMs continuously increased during the incubation period, suggesting good proliferation and interaction between the cells and NFMs. Cells grown on random PLGA NFMs exhibited random adhesion and growth. In contrast, the cells on aligned NFMs were found to have elongated their actin microfilaments 1 d after seeding, but more importantly, the direction of their elongation was the same as that of the aligned nanofibers from the initial phase of their adherence, compared to the randomly oriented cells on random PLGA NFMs (Figure 4A,D). By day 3 after cell seeding, most cells had attached and elongated themselves along the direction of the aligned nanofibers with a spindle-shaped appearance. Although the direction of the elongated NIH-3T3 cells was not identical to that of the nanofibers, a distinct regularity was identified throughout the incubation period when compared with the randomly oriented cells on random PLGA NFMs (Figure 4B,E). On day 7, the cells completely covered the surface of the NFM, suggesting that the actin filaments in NIH-3T3 cells had aligned along the aligned nanofibrous scaffold (Figure 4F). Furthermore, aligned NFMs presented a specific topographical cue to orient the cells, thereby avoiding aggregation at the end of day 7. In contrast, the cells on random NFMs were likely to adhere to each other, forming an amorphous aggregate rather than attaching to the nanofibers (Figure 4C).

### 3.5. Cell Attachment and Proliferation 

**Figure 5:**
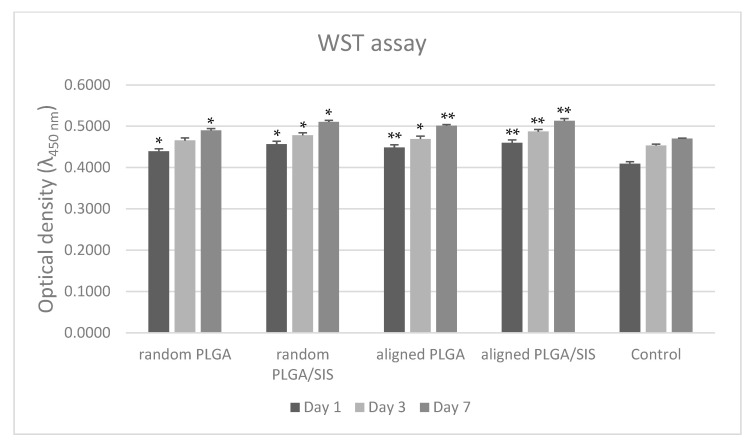
Cell viability of NIH-3T3 cells seeded on electrospun random PLGA, aligned PLGA, random PLGA/SIS, and aligned PLGA/SIS was measured by formazan absorption at 450 nm in WST assay (*n* = 6). (Comparison with control, *—*p* < 0.05, **—*p* < 0.001).

We evaluated the effects of nanofiber alignment on the initial cell attachment and proliferation by seeding NIH-3T3 cells in random PLGA, random PLGA/SIS NFMs, aligned PLGA, and aligned PLGA/SIS NFMs. Cell attachment and proliferation was evaluated using a WST assay kit after days 1, 3, and 7 (Figure 5). An absorbance at 450 nm indicated that random PLGA/SIS had a higher optical density (OD) value than aligned PLGA, with significant statistical differences (*p* < 0.05). This suggests that the increase in hydrophilicity due to incorporation of SIS into PLGA is more effective in inducing cell proliferation than the alignment of the nanofibers. In contrast, aligned PLGA/SIS had a higher OD value than random PLGA/SIS (*p* < 0.05). It is speculated that the longitudinal alignment of the nanofibers guided the spreading direction of the attached cells, thereby allowing the cells to occupy the space more compactly. Thus, more cells were deposited within the surface of the scaffolds. Moreover, according to a previous study, the degradation rate of aligned nanofibers is lower than that of random nanofibers [34]. Considering the profound effect of alignment of the nanofibers on cell proliferation, maintenance of their morphology would be beneficial for cellular growth.

## 4. Conclusions

In this study, PLGA/SIS NFMs that were aligned with a rotational wheel collector were fabricated using electrospinning for potential use in tendon engineering. We demonstrated that the mechanical properties of the aligned PLGA/SIS NFMs were superior to those of the random PLGA, random PLGA/SIS, or aligned PLGA NFMs. Furthermore, the cellular behavior of aligned PLGA/SIS nanofibers was more effective than that of random PLGA or random PLGA/SIS NFMs, allowing more cells to be deposited above the surface of the scaffolds. Consequently, the aligned PLGA/SIS NFMs facilitated increased cell adhesion and proliferation when compared with random PLGA, random PLGA/SIS, or aligned PLGA NFMs due to the regular orientation of nanofibers or SIS incorporation into PLGA. Considering its mechanical properties and cellular behavior, aligned PLGA/SIS is a promising candidate for tendon tissue engineering.

## Figures and Tables

**Table 1:** Measurements of the mechanical properties of each scaffold (*n* = 6). All data sets are significantly different from each other (*p* < 0.05).

		Elastic Modulus (MPa)	Ultimate Tensile Strength (MPa)	Break Strain (%)
Random	PLGA	212.01 ± 25.25	3.91 ± 0.98	18.23 ± 3.87
	PLGA/SIS	351.87 ± 15.79	7.12 ± 1.22	31.43 ± 4.98
Aligned	PLGA	420.59 ± 49.84	9.51 ± 1.13	7.92 ± 2.91
	PLGA/SIS	520.69 ± 54.11	15.14 ± 4.12	22.71 ± 5.12

## Data Availability

The data presented in this study are available upon request from the corresponding authors.
